# Electrophysiologic Mapping of the Extraocular Motor Nuclei

**DOI:** 10.7759/cureus.16587

**Published:** 2021-07-23

**Authors:** Justin W Silverstein, Jason A Ellis

**Affiliations:** 1 Neurology, Lenox Hill Hospital Northwell Health, New York, USA; 2 Neurology, Neuro Protective Solutions, New York, USA; 3 Neurosurgery, Lenox Hill Hospital Northwell Health, New York, USA

**Keywords:** neuro-monitoring, neuro-surgery, skull base surgery, cranial nerves, extraocular

## Abstract

Mapping the floor of the fourth ventricle to identify the motonuclei of cranial nerves VII-XII has been well-described. Though there are some reports of stimulating the pontomesencephalic surface to identify the extraocular motor nuclei, there is a debate as to its efficacy and utility in helping to identify safe entry zones for medullary incision in an intra-axial resection. We present two cases where we positively and negatively mapped the surface of the midbrain and rostral pons to assist in surgical decision-making. Both patients had gross total resections of cavernomas, and both awoke without any new onset extraocular motor deficits.

## Introduction

Cranial nerve (CN) monitoring and mapping is routine practice during many skull base procedures. The electrophysiological identification of neural structures when the anatomy is distorted due to a lesion is paramount in reducing postoperative morbidity. Intra-axial lesions may distort and displace anatomical landmarks, as well as fiber tracts and CN nuclei. The use of electrophysiological stimulation of CN nuclei has been shown to be effective in gaining safe entry to intra-axial pontine lesions; however, these techniques are still debated when describing lesions within the midbrain [1-3]. We present two cases where we positive mapped and negative mapped the midbrain and upper pons for safe entry to pontomesencephalic lesions.

## Case presentation

Neurophysiological monitoring and mapping methods

For both procedures, a multimodal neuromonitoring paradigm was elected for use, which included spontaneous electromyography (EMG) of cranial nerves III, IV, VI, & VII (CN EMG), brainstem auditory evoked potentials (BAEP), cranial nerve motor evoked potentials (CNMEP), motor-evoked potentials (MEP) with long-tract recordings (LTMEP), somatosensory evoked potentials (SSEP), and direct cortical MEP (DCMEP) (second case only).

CN EMG

**Figure 1:**
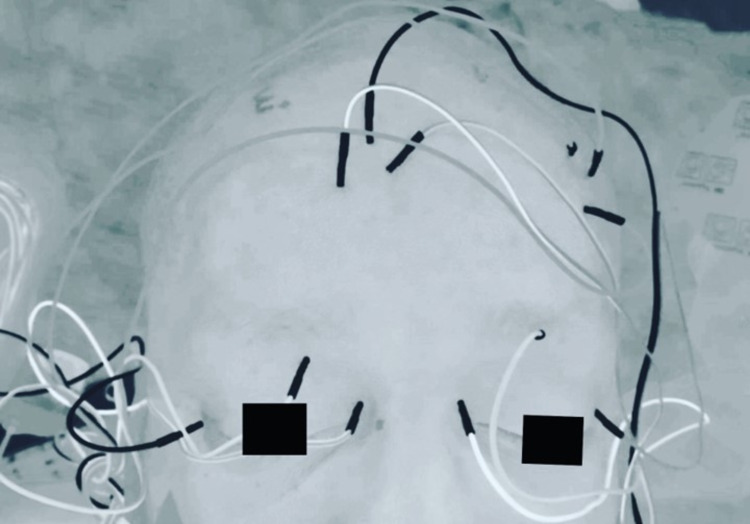
Extraocular EMG setup Needle electrodes placed in the medial rectus, superior oblique, and lateral rectus muscles bilaterally. These needles are referenced to needles placed in the opposite forehead for each eye. EMG: electromyography

The medial rectus muscle, superior oblique, and lateral rectus muscles were targeted bilaterally for CN III, IV, and VI coverage, respectively (Figure 1). A referential EMG montage was elected for use with a single needle electrode placed in the extraocular muscles in the intraorbital space bilaterally referenced to electrodes placed on the forehead as described by previous authors [4]. The extraocular EMG electrodes were placed by a supervising doctorate-level neurophysiologist. When placing the electrodes for the oculomotor, trochlear, and abducens nerves, care was taken to avoid the globe of the eye. With the assistance of the anesthesiologist, the eyes were protected with Tegaderm (3M Company, Saint Paul, Minnesota), and avoidance of excess pressure caused by neuromonitoring leads was appreciated. For CN VII coverage, we targeted the frontalis, orbicularis oculi, orbicularis oris, and mentalis muscles using a bipolar montage bilaterally with standard intraoperative EMG electrodes.

CNMEP

Stimulation electrodes were placed on the scalp at Mz and M3/M4 as described by Dong et al. [5]. A train of five pulses was utilized with an interstimulus interval of 1 ms, a pulse width of 50 µsec, and a stimulus intensity up to 300V. The train of five pulses was reduced to a single pulse to demonstrate an absence of peripheral responses to stimulation to distinguish between central conduction and peripheral conduction.

BAEP

Foam ear inserts were introduced to the external auditory meatus binaurally to deliver click stimulation at a rep rate of 11.1 Hz. Recording electrodes were placed on the ear lobe and at Cz of the international 10-20 system. Monoaural stimulation was presented at 90 db using alternating (rarefaction and condensation) clicks with a contemporaneous monoaural masking noise delivered at 40 db to the non-stimulated ear.

SSEP

SSEP stimulation and recording were delivered in a standard fashion. The median, ulnar, and posterior tibial nerves were evaluated throughout the procedure.

LTMEP

Standard stimulation parameters were used for the activation of the corticospinal tracts. Bilateral upper extremity and lower extremity muscles were used to record LTMEP compound muscle action potentials (CMAP) using standard intraoperative EMG electrodes.

During the second case, we elected to place a 1x8 subdural strip electrode to monitor the functional integrity of the corticospinal tracts (CSTs) via direct cortical stimulation. A train of five with a 500 Hz pulse width was used for DCMEP.

Results

Case One

**Figure 2:**
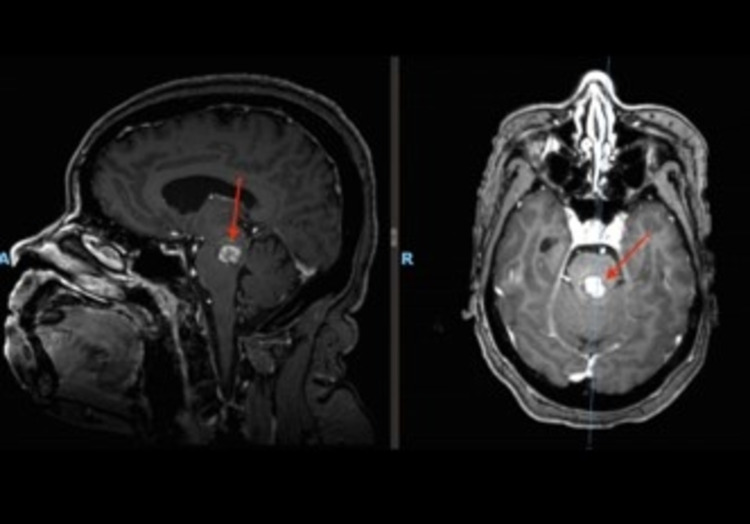
Case 1: axial and sagittal MRI revealing an intra-axial midbrain cavernoma

A 53-year female was transferred to our institution after experiencing multiple symptomatic brainstem hemorrhages resulting in progressive ophthalmoplegia and quadriparesis over the course of two weeks. She was ventilator dependent, disoriented, unable to communicate, and restless. A brain magnetic resonance image (MRI) revealed a ruptured dorsal midbrain cavernoma (Figure 2). Due to her deteriorating status, a microsurgical resection of the cavernoma was elected.

**Figure 3:**
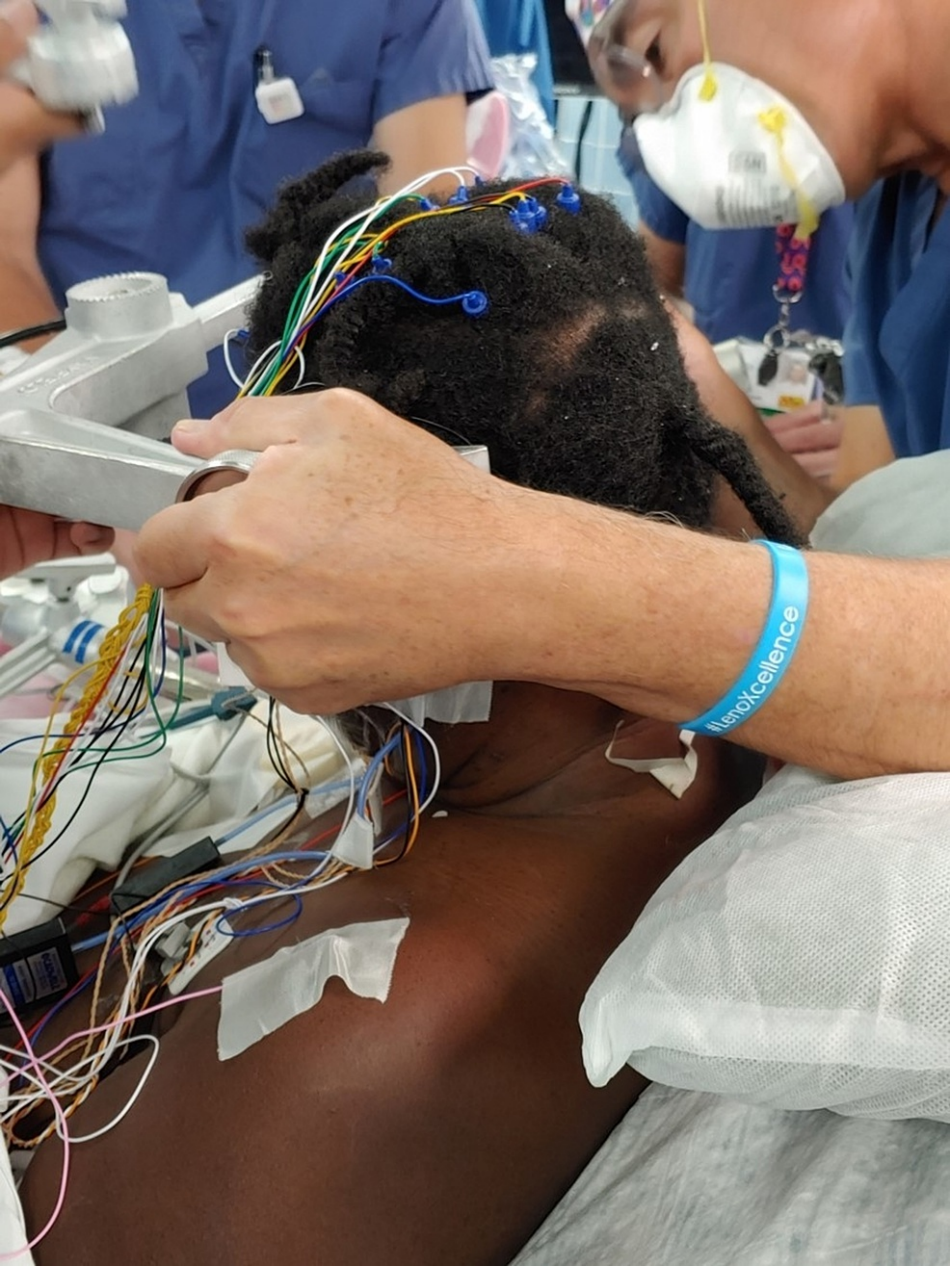
Patient in a sitting position with neuromonitoring leads affixed

A midline suboccipital craniotomy was performed via a supracerebellar infratentorial approach in a seated position under exoscopic 3D magnification (Olympus Orbeye Exoscope, Center Valley, PA) with endoscopic evaluation. The patient was positioned in a sitting position with her head fixated by a three-point skull clamp (Figure 3). Electrodes used for neurophysiological monitoring were applied prior to skin incision.

**Figure 4:**
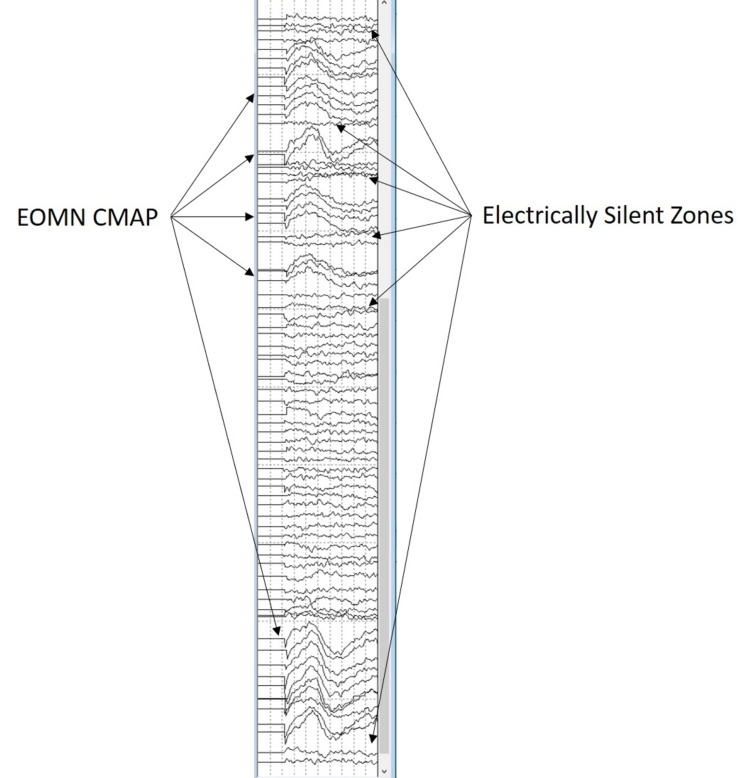
Compound muscle action potential (CMAP) from the medial rectus muscles during surface stimulation of the midbrain in Case 1 Notice the areas of activation vs the area where no CMAP is appreciated.

**Figure 5:**
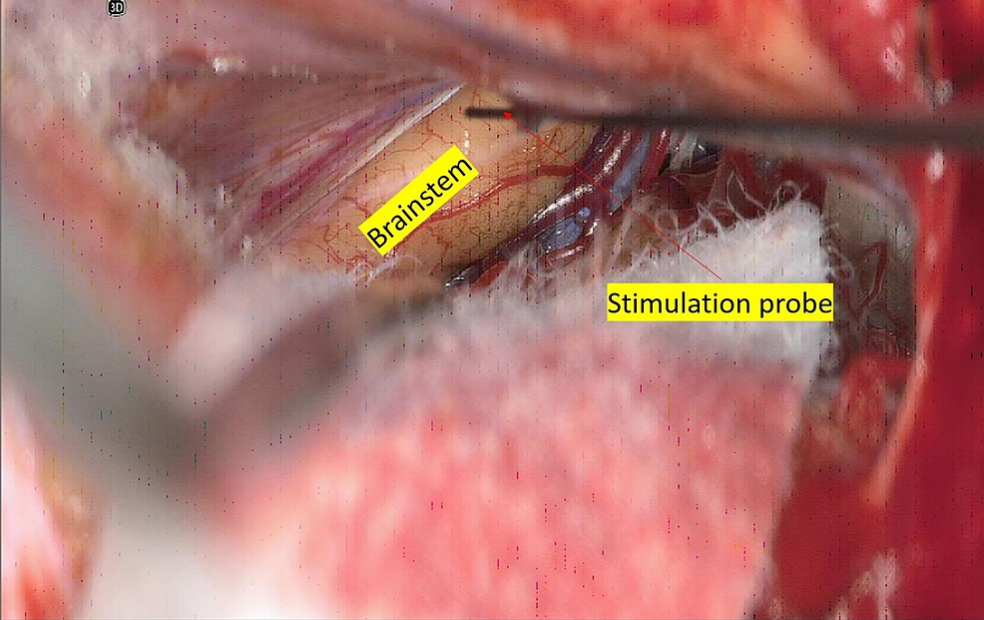
Monopolar electric stimulation of the brainstem

Prior to skin incision, baseline recordings were obtained from the neuromonitoring modalities used. SSEP and LTMEP were obtained from all extremities. The BAEP was only obtained from the left side, and CNMEP was only obtained from facial nerve innervated muscles. After the suboccipital craniotomy was performed and the dura opened, a supracerebellar/infratentorial corridor was created by cauterizing a single tentorial bridging vein. Next, the arachnoid within the pineal region and quadrigeminal cistern was dissected on approach to the dorsal midbrain. Once at the midbrain, a flush tip monopolar stimulating probe was used to stimulate the dorsal mesencephalon. Stimulation was set to 0.3 mA, and we were adequately able to identify the approximate region of the midline oculomotor nuclei (OMN). Leaving the stimulus at this level, we also identified areas of the midbrain that were electrically silent (Figures 4-5). The surgeon used positive activation of the OMN as their guide to safely enter the brainstem. A gross total resection of the cavernoma was successfully performed and confirmed with endoscopic evaluation. At four months postoperatively, the patient is alert and moving all extremities. The patient’s extraocular muscles are intact; however, the patient does have persistent internuclear ophthalmoplegia.

Case Two

**Figure 6:**
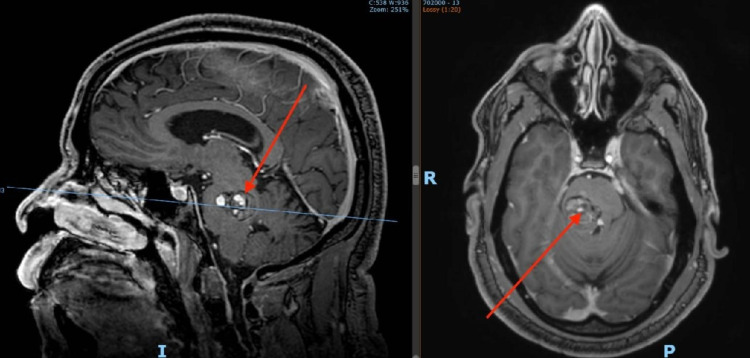
Axial and sagittal views of a pontomesenphalic cavernous malformation (Case 2)

A 53-year-old male experienced a hemorrhagic stroke and was admitted to our facility with progressive left-sided hemiparesis and hemiparesthesia. MRI shows a ruptured midbrain-pontine cavernoma, eccentric to the right side (Figure 6). He was taken to the operating room for complete resection.

**Figure 7:**
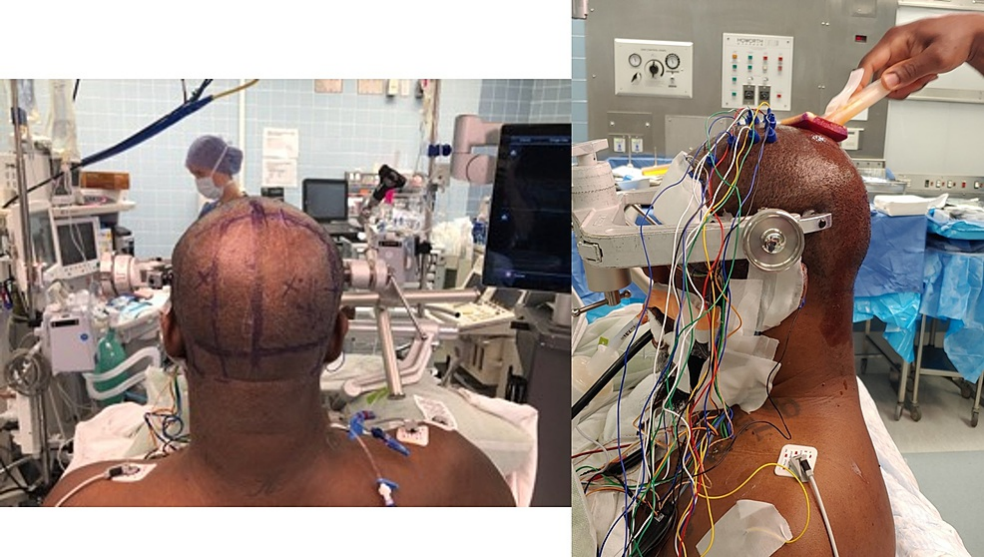
The patient was positioned in a sitting position with his head fixated by a three-point skull clamp with neuromonitoring leads affixed

The patient underwent a craniotomy via an occipital-transtentorial approach to the dorsolateral pons under exoscopic 3D magnification (Olympus Orbeye Exoscope, Center Valley, PA) with endoscopic evaluation. The patient was positioned in a sitting position with his head fixated by a three-point skull clamp (Figure 7). Electrodes used for neurophysiological monitoring were applied prior to skin incision.

Prior to skin incision, baseline recordings were obtained from the neuromonitoring modalities used. SSEPs were only obtained from the right extremities. Transcranial MEPs (TCMEPs) were obtained in the bilateral upper extremities only, with the left upper extremity having a markedly lower amplitude compared to the right upper extremity. The BAEP was obtained binaurally, and CNMEP was not obtained due to peripheral activation.

After the occipital craniotomy was performed and the dura opened, the tentorial incisura was cut to provide a corridor from the supra- to the infra-tentorial compartment. Once at the brainstem, an electrified monopolar ring dissector was used to search for extraocular motor nuclei (EOMN). Stimulation was set to 0.5 mA. Different areas of the brainstem were stimulated, and no CMAPs were appreciated. The intensity was then increased to 0.8 mA, and the same areas were stimulated again with no response noted. The surgeon used the negative activation of the EOMN as their guide to safely enter the brainstem. A gross total resection of the cavernoma was successfully performed and confirmed with endoscopic evaluation. The patient is neurologically stable with intact extraocular motor function postoperatively at one month.

## Discussion

The oculomotor complex is a complicated system consisting of four subsets of nuclei that are situated near the dorsomedial part of the tegmentum [6-8]. The most medial subnuclei innervate the superior rectus muscles, where the remaining subnuclei have axons that extend to the inferior rectus muscles. The neurons supplying the medial rectus muscles are distributed into three separate subnuclei [7]. The trochlear nuclei are found midline and ventral to the cerebral aqueduct in the midbrain at the level of the inferior colliculus, whereas the abducens nuclei are found in the caudal pons beneath the facial colliculus [6].

Stimulating the rhomboid fossa at the floor of the fourth ventricle has been described to map the nuclei for cranial nerves VII through XII in order to find safe entry zones into the brainstem for intra-axial lesions [1,3-4]. Duffau and Sichez first described using electrical stimulation for a midbrain lesion in 1998; however, they did not use EMG recordings but instead used a 60 Hz low frequency, long train bipolar stimulation at the level of the inferior colliculus [2]. They visualized an elevation of the eye during stimulation and used the area where no elevation was noted to make their medullary incision. Eisner et al. presented 16 cases where they mapped the motor nuclei of the brainstem using electrical stimulation and recording from muscles with EMG electrodes placed [3]. They determined which nerves to target based on where the space-occupying lesion was. They describe targeting the extraocular cranial nerves (EOCN); however, none of their lesions were in the midbrain. Interestingly, they did activate the oculomotor system when stimulating cranially and medially to the facial genu. Ishihara et al. did not activate the EOMN with surface stimulation of the midbrain in any of their four cases [6]. They did, however, elicit CMAPs from the EOMN within the cavity of the lesions and used those areas of positive activation as areas to avoid during resection. They argue that the techniques used in pontine lesions for surface stimulation to identify a safe entry zone are not reliable in midbrain lesions, and surgeons should use anatomical landmarks for medullary incisions instead.

## Conclusions

In our illustrative case report, we were able to identify the OMN in one case with surface stimulation and not the other. The surgeon used the information from the electrically silent areas in both cases to make the medullary incision. Both patients awoke with no new extraocular motor deficits. These procedures are very rare, and there are no large case series that describe these techniques specifically for midbrain and upper pontine lesions. More research is needed with larger cohorts in order to establish statistical efficacy.
